# Bacterial profile and drug susceptibility among adult patients with community acquired lower respiratory tract infection at tertiary hospital, Southern Ethiopia

**DOI:** 10.1186/s12879-021-06151-2

**Published:** 2021-05-13

**Authors:** Alemitu Beyene Gebre, Tsegaye Alemayehu Begashaw, Moges Desta Ormago

**Affiliations:** Hawassa University College of Medicine and Health Science, Hawassa, Ethiopia

**Keywords:** Lower respiratory tract infection, Culture-positive, Antimicrobial susceptibility test

## Abstract

**Background:**

Lower respiratory tract infection is a global problem accounting over 50 million deaths annually. Here, we determined the bacterial profile and antimicrobial susceptibility pattern of lower respiratory tract infections among adult patients attending at Tertiary Hospital, Southern Ethiopia.

**Methods:**

A cross sectional study was conducted among adult patients with lower respiratory infection at the medical outpatient department of the Hospital. A sputum sample was collected and processed for bacterial culture and antimicrobial susceptibility test. Semi structured questionnaires were used to collect data. SPSS version 22 software was used for statistical analysis and a *p* value of < 0.05 was considered as statistically significant.

**Results:**

Out of 406 sputum samples of participants 136(33.5%) were culture positive for 142 bacterial isolates. *Klebsiella pneumoniae* 36(25.4%) was the predominant isolate followed by *Pseudomonas* species 25(17.6%). Gram-negative bacteria were sensitive to cefepime (86.0%) and ciprofloxacin (77.8%) antibiotics while gram-positive (76.5%) to clindamycin.

**Conclusion:**

Community acquired lower respiratory tract Infection was highly prevalent in the study area and the isolates showed resistant to common antibiotics such as ampicillin, augmentin, ceftazidime and tetracycline. Therefore, culture and susceptibility test is vital for appropriate management of lower respiratory tract infection in the study area.

**Supplementary Information:**

The online version contains supplementary material available at 10.1186/s12879-021-06151-2.

## Background

Lower respiratory tract infections (LRTIs) are one of the main respiratory diseases that challenging the world and they remain the deadliest communicable disease and the 3rd leading cause of death around the world, after ischaemic heart and cerebrovascular diseases [1]. In Sub-Saharan Africa, LRTIs rank third after HIV/AIDS and malaria in terms of causes of mortality [2].

The etiological agents of LRTI infections vary between populations and countries, depending on the difference in geography, climate, and socioeconomic conditions, associated factors of LRTI as well as their antibiotics susceptibility [3].

Community acquired pneumonia (CAP) an acute infection of the pulmonary parenchyma occurring in a patient who has not resided in a hospital or health care facility for greater than 14 days before onset of symptoms. The initial antimicrobial management for community acquired pneumonia is usually empirical and selecting an appropriate regimen requires knowledge of the spectrum of organisms implicated in CAP locally [4]. Therefore, studies are critical to identify the microorganisms causing LRTI in the local context and to determine their susceptibility to various antimicrobials. Initial empirical broad-spectrum therapy can then be narrowed based on the culture results [5]. This study aimed to determine the bacterial pathogens with their antimicrobial susceptibility pattern and associated factors in adult patients with LRTI.

## Method

### Study design, period and population

Cross-sectional study was conducted in Hawassa University Comprehensive Specialized Hospital (HUCSH), Southern Ethiopia from July to October 2019. Individual patients with age of ≥18 years old having symptoms of LRTI, in particular, productive cough, fever, chest pain and acid-fast bacilli (AFB) smear-negative were included in the study. Patients who were on antibiotic treatment in the last 14 days, patients who were experienced tuberculosis in the last 2 years, those who were severely ill and unable to give sputum sample and those who were failed the Bartlett’s criteria were excluded.

### Sample size determination and sampling technique

The sample size was determined using single proportion formula,

$$ \mathrm{n}={\left({\mathrm{z}}_{\upalpha /2}\right)}^2\left(\mathrm{p}\ \left(1-\mathrm{p}\right)\right)/{\mathrm{d}}^2 $$

considering the 95% confidence level CI (z = 1.96) taking the prevalence (p) as 40% from previous study conducted in Arba Minch [6] and margin of error (5%), (d = 0.05) the formula used to calculate the sample size (n) was as follow.

$$ \mathrm{n}=\frac{(1.96)2\ \left(0.4\left(1\hbox{-} 0.4\right)\right)\ }{0.05^2}=369 $$

With contingency of 10% = 369 + 37 = 406.

Systematic random sampling technique was applied to select 406 respondents for interview. The interval (K) was obtained by dividing the total number of patients attending the medical OPD for LRTI during the study period by sample size.

According to information found from the data base of HUCSH the average daily patient flow due to LRTI at OPD is 11. The study period took 3 months, which contain 66 days and 11 times 66 gives 726. Therefore, the total patient flow during the study period is 726.

$$ \mathrm{K}=726/406=1.8\approx 2 $$

The first individual is selected by lottery method and the others at regular interval.

### Data collection and laboratory processing

All relevant socio-demographic and clinical data were collected by trained nurses through face-to-face interviews with the patient using a semi structured questionnaire from 406 respondents whose sputum were AFB smear-negative and fulfill Bartlett’s criteria on Gram’s stain. Spot- spot sputum samples were collected with one-hour interval using dry, sterile, leak proof, translucent, and screw-capped plastic containers with a capacity of 30 ml and brought to the Microbiology Laboratory of HUCSH for laboratory processing.

### Sputum microscopy for AFB

All Sputum samples were examined by the Light emitting diode fluorescence microscope (LED FM) for AFB detection [7]. The AFB appeared as bright yellow against dark back ground materials. Sputum smear positive cases were excluded from the study whereas sputum smear negative cases were evaluated further based on Bartlett’s grading system and macroscopically [8].

### Gram’s stain

Their sputum samples were checked macroscopically for color, volume, viscosity, odor, and any positive score (sum of + and – values assigned) on Gram’s stain was considered as acceptable result to culture.

Sputum sample based on Bartlett’s criteria [8].

### Cultivation and identification of isolates

The purulent part of accepted sputum sample was inoculated to blood agar (Oxoid, Hampshire, UK), MacConkey agar (Oxoid, Hampshire, UK) and chocolate agar (Oxoid, Hampshire, UK) with the sterile wire loop. In order to get a single pure colony, the samples were streaked into four quadrants of the plate with flaming the loop in between each spread. The inoculated MacConkey agar plates were incubated aerobically at 37 °C for 24 h whereas blood and chocolate agar were inoculated using 5–10% CO_2_ generating candle jar at 37 °C for 24 h. After 24 h incubation, the plate was examined for growth. Pure colonies were sub-cultured to nutrient broth (Oxoid, Hampshire, UK). Finally, bacterial species were identified by using the standard microbiological technique [9].

Pure isolates of bacterial pathogens were preliminarily characterized by colony morphology, gram stain and hemolytic reactions on blood agar plates. Identification of bacteria down to species level was done by biochemical tests such as catalase, coagulase, optochin (30 μg) test, bacitracin (30 μg) test and bile esculin agar for Gram-positive identification and oxidase, indole production, urease, citrate utilization, lysine decarboxylation, carbohydrate fermentation, gas production, H_2_S production and motility for Gram-negative bacteria [10].

### Antimicrobial susceptibility testing

Antimicrobial susceptibility test (AST) was performed for identified bacterial isolates by disc diffusion technique as recommended by Clinical and Laboratory Standard Institute on Mueller-Hinton agar (Oxoid, Hampshire, UK) and 5% sheep blood supplemented Mueller-Hinton agar for fastidious bacterial isolates. Based on CLSI (2019) guideline the following antibiotics were used for this study: gentamycin-GN (10 μg), erythromycin-ERY (15 μg), ciprofloxacin-CPR (5 μg), tetracycline-TAT (30 μg), ampicillin-AMP (10 μg), augmentin-AUG (10 μg), ceftriaxone-CTR (30 μg), chloramphenicol-CAF (30 μg), ceftazidime-CAZ (10 μg), cotrimoxazole-COT (1.25/23.75 μg), cefepime-CEP (30 μg), cefoxitin-CXT (30 μg), penicillin-PEP (10 μg), clindamycin-CLD (2 μg) and. Finally, the result was reported as sensitive (S), intermediate (I) or resistance (R) by measuring the diameter of zone of inhibition or hemolysis [11].

### Quality control

Prior to actual data collection, the quality of data was assured by pre-testing questionnaires on 20 participants at HUCSH for assessing its clarity and to take amendments and orientation was given to data collectors. The prepared culture media were checked for sterility by incubating the 5 % of prepared media for 24 h and observed for the presence of any colony growth. In addition, the abilities of the prepared media supporting the growth of organisms were checked by inoculating control strains. Standard reference strains of *S. pneumoniae* (ATCC-49619), *H. influenzae* (ATCC-49766) *E. coli* (ATCC-25922), *S. aureus* (ATCC-25923), *P. aeruginosa* (ATCC-27853) and *P. mirabilis* (ATCC-12453) were used during culture and antimicrobial susceptibility testing.

### Data processing and analysis

All filled questionnaires for this study was checked visually, coded and entered into excel and then exported to SPSS version22 software (SPSS Inc., Chicago, IL, USA) for analysis. Bivariate logistic regression was used to determine predictors of culture confirmed LRTI. For those variables, which *p*-value < 0.25 in the bivariate, the analysis was further entered into the multivariable logistic regression model [12]. Associations between dependent and independent variables were assessed and its strength was described using odds ratios at 95% confidence intervals. A statistically significant association considered as *p*-value < 0.05.

## Result

### Sociodemographic characteristics

A total of 406 LRTI suspected adult patients were enrolled in this study. Of them, 246(60.6%) were male. The mean age of patients was 36.75 ± 14.84. More than half of the participants live in urban 225(55.4%) [Table 1].

**Table 1:** Sociodemographic characteristics of adult patients with lower respiratory tract infection at HUCSH, 2019

Variables		Frequency	Percent (%)
Sex	Male	246	60.6
	Female	160	39.4
Age (year)	18–35	235	57.9
	36–49	93	22.9
	50–64	51	12.6
	> 65	27	6.7
Residence	Rural	181	44.6
	Urban	225	55.4
Education status	No formal education	142	35.0
	1–8 grade	141	34.7
	9–12 grade	70	17.2
	Diploma & above	53	13.1
Marital status	Single	104	25.6
	Married	302	74.4
Occupation	Farmer	89	21.9
	Government	43	10.6
	Private	93	22.9
	Housewife	112	27.6
	Student	52	12.8
	Jobless	17	4.2
Monthly income (Ethiopian birr)	< 1000	126	31.0
	1000–2000	79	19.5
	2001–3000	118	29.1
	3001–5000	52	12.8
	> 5000	19	4.7
	No monthly income	12	3.0

### Clinical characteristics

Among 406 study participants, twenty six (6.4%) of participants have previous tuberculosis disease exposures before 2 years and 34(8.4%) of participants have heart disease and 26(6.4%) were HIV positive [Table 2].

**Table 2:** Clinical characteristics of adult patients with lower respiratory tract infection at HUCSH, 2019

Variables		Frequency	Percent (%)
Cigarette smoking	Smoker	33	8.1
	No smoker	373	91.9
Alcohol drinking	Yes	55	13.5
	No	351	86.5
Crowded living condition	Yes	52	12.8
	No	354	87.2
History of the previous TB	Yes	26	6.4
	No	380	93.6
Heart disease	Yes	34	8.4
	No	372	91.6
Previous HIV screening	Positive	26	6.4
	Negative	167	42.1
	Unscreened	213	52.5

### Bacterial profile of LRTIs

The overall culture-positive sputum sample from a patient with LRTI in this study were 136/406(33.5%), 95% CI (28.8–40%) with a total bacterial isolate of 142/406 (35.0%). Single bacterial pathogens were isolated from 130(32.0%) patients, while mixed infections were isolated from 6(1.4%) patients. Among mixed infection isolate, *E. coli* and *S. aureus* were isolated from 1(0.2%) patient, *E. coli* and *Pseudomonas* spp. from 1(0.2%) patient and *K. pneumoniae* and *E. coli* from 4(1.0%) patients. The gram-negative bacteria 108(76.0%) predominated over gram-positive bacteria. In this study, *K. pneumoniae* 36(25.4%) was the frequently isolated bacteria followed by *Pseudomonas* spp. 25(17.6%), *E. coli* 22(15.5%) and *S. aureus* 21(14.8%) [Table 3].

**Table 3:** Frequency of bacterial isolates identified from sputum specimen of an adult patient with community acquired LRTI at HUCSH 2019

Isolates	Frequency	Percent (%)
*K. pneumoniae*	36	25.4
*Pseudomonas* spp	25	17.6
*E. coli*	22	15.5
*S. aureus*	21	14.8
*Acinetobacter spp*	19	13.4
*S.pneumoniae*	9	6.3
*Enterobacter spp*	4	2.8
*Others*^a^	6	4.2

^a^*Citrobacter spp, S.pyogene and Enterococcus spp*

The bacterial pathogens were more predominant in male 87(64.0%) than female as the study result shows. The age group 18–35 years was more susceptible to bacterial pathogens of LRTI 64(47.1%) than other age groups. Of 302 married study participants enrolled in this study, 112(82.4%) were positive for sputum culture. Among 43(10.6%) government workers participated in study 11(8.1%) were positive for bacterial pathogens of LRTI.

In bivariate analysis the variables; age range of 18–35 years (COR (crude odds ratio) =3.886, 95% CI, 1.712–8.821, *p* = .001) and 36–49 years (COR = 2.773, 95% CI, 1.151–6.677, *p* = .023); educational status who were 1–8 grade students (COR = .596, 95% CI, .296–1.199, *P* = .147), marital status those were married (COR = .509, 95% CI, .305–.850, *P* = .010), monthly income with 2001–3000 (COR = .336, 95% CI, .070–1.606, *P* = .172) and > 5000 (COR = .222, 95%(.038–1.298, *P* = .095). In addition those who were non-cigarette smoker (COR = 1.984, 95% CI, .969–4.062, *P* = .061), non-alcohol drinker (COR = 1.652, 95%CI, .927–2.945, *P* = .089), had no chronic heart disease (COR = 1.638, 95% CI, .804–3.334, *P* = .174) and HIV result of negative (COR = .749, 95% CI, .486–1.154, *P* = .190), participants who were unscreened for HIV at all (COR = .420, 95%CI, .184–.957, *P* = .039) were candidate variable for multivariate analysis with *p*-value < .250.

However, in multivariate analysis the age range 18–35 years (AOR (adjusted odds ratio) = 3.856, 95% CI, 1.584–9.387, *P* = .003), 36–49 years (AOR = 3.136, 95% CI, 1.247–7.888, *P* = .015), marital status those who are married (AOR = .450, 95% CI, .207–.980, *p* = .044), occupation in government (AOR = 6.303, 95% CI, 1.508–26.341, *P* = .012) and monthly income > 5000 (AOR = .060, 95% CI, .007–.526, *P* = .011) were the only statically significant associated risk factors for LRTI [Table 4].

**Table 4:** Associated factors of culture-positive sputum sample of a patient with community acquired LRTI at HUCSH, 2019 (*n* = 406)

Variables		Growth		COR (95%CI)	*P*-value	AOR (95%CI)	*P*-value
		Yes (%)	No (%)				
Sex	Male	87 (64.0)	159 (58.9)	.807(.527–1.235)	0.323		
	Female	49 (36.0)	111 (41.1)	1			
Age (year)	18–35	64 (47.1)	171 (63.3)	3.886 (1.712–8.821)	0.001	3.856 (1.584–9.387)	0.003*
	36–49	32 (23.5)	61 (22.6)	2.773 (1.151–6.677)	0.023	3.136 (1.247–7.888)	0.015*
	50–64	24 (17.6)	27 (10.0)	1.636(.636–4.207)	0.307	1.717(.637–4.631)	0.285
	> 65	16 (11.6)	11 (4.1)	1			
Residence	Rural	62 (45.6)	119 (44.1)	1			
	Urban	74 (54.4)	151 (55.9)	.772(.703–1.609)	0.772		
Educational status	No formal education	48 (35.3)	94 (34.8)	.703(.348–1.419)	0.326	.613(.194–1.931)	0.403
	1–8 grade	53 (39.0)	88 (32.6)	.596(.296–1.199)	0.147	.427(.146–1.248)	0.120
	9–12 grade	21 (15.4)	49 (18.1)	.838(.378–1.857)	0.663	.556(.188–1.646)	0.289
	Diploma & above	14 (10.3)	39 (14.4)	1			
Marital status	Single	24 (17.6)	80 (29.6)	1			
	Married	112 (82.4)	190 (70.4)	.509(.305–.850)	0.010	.450(.207–.980)	0.044^*^
Occupation	Farmer	31 (22.8)	58 (21.5)	.689(.325–1.462	0.332	2.661(.841–8.413)	0.096
	Government	11 (8.1)	32 (11.9)	1.072(.428–2.687)	0.882	6.303 (1.508–26.341)	0.012^*^
	Private	34 (25.0)	59 (21.9)	.639(.304–1.345)	0.239	2.425(.882–6.668)	0.086
	Housewife	41 (30.1)	71 (26.3)	.638(.309–1.315)	0.223	2.048(.656–6.392)	0.217
	Student	14 (10.3)	38 (14.1)	.884(.264–2.965)	0.842	3.192(.709–14.365)	0.131
	Jobless	5 (3.7)	12 (4.4)	1			
Monthly income	< 1000	38 (27.9)	88 (32.6)	.463(.097–2.215)	0.335	.461(.083–2.551)	0.375
	1000–2000	26 (19.1)	53 (19.6)	.408(.083–1.997)	0.268	.443(.076–2.578)	0.365
	2001–3000	44 (32.4)	74 (27.4)	.336(.070–1.606)	0.172	.310(.055–1.752)	0.185
	3001–5000	17 (12.5)	35 (13.0)	.412(.081–2.091)	0.285	.265(.043–1.61)	0.150
	> 5000	9 (6.6)	10 (3.7)	.222(.038–1.298)	0.095	.060(.007–.526)	0.011
	No monthly income	2 (1.5)	10 (3.7)	1			
Cigarette Smoking	Smoker	16 (11.8)	17 (6.3)	1			
	No smoker	120 (88.4)	253 (93.7)	1.984(.969–4.062)	0.061	2.274(.810–6.385)	0.119
Alcohol drinking	Yes	24 (17.6)	31 (11.5)	1			
	No	112 (82.4)	239 (88.5)	1.652(.927–2.945)	0.089	1.112(.468–2.642)	0.809
Crowded living condition	Yes	16 (11.8)	36 (13.3)	1			
	No	120 (88.2)	234 (86.7)	1.154(.615–2.164)	0.655		
History of previous TB	Yes	9 (6.6)	17 (6.3)	1			
	No	127 (93.4)	253 (93.7)	1.055(.457–2.432)	0.901		
Chronic heart disease	Yes	15 (11.0)	19 (7.0)	1			
	No	121 (89.0)	251 (93.0)	1.638(.804–3.334)	0.174	1.530(.688–3.401)	0.297
Previous HIV screening	Positive	13 (9.6)	13 (4.8)	1			
	Negative	60 (44.1)	107 (39.6)	.749(.486–1.154)	0.190	.524(.202–1.358)	0.183
	Unscreened	63 (46.3)	150 (55.6)	.420(.184–.957)	0.039	1.301(.803–2.110)	0.285

*CI* Confidence Interval, *OR* Odds Ratio

### Antimicrobial susceptibility patterns of bacterial isolates

#### Gram positive bacteria

In this study, Gram-positive were sensitive to clindamycin 26(76.5%) and erythromycin 19(55.9%) however, they showed resistant to tetracycline 20(58.8%). *S. aureus* was sensitive to cefoxitin 19(90.6%), gentamycin 16(76.2%) and ciprofloxacin 15(71.4%). *S. aureus* was resistant to tetracycline 13(61.9%) and cotrimoxazole 10(47.6%). *S. pneumoniae* were sensitive to clindamycin 8(88.9%), penicillin 7(77.8%), erythromycin 6(66.7%), but *S. pneumoniae* was resistant to tetracycline 6(66.7%). All *S. pyogenes* isolates were 3(100%) sensitive to tetracycline, ampicillin, clindamycin, erythromycin and penicillin [Table 5].

**Table 5:** Antibiotic susceptibility patterns of gram-positive bacterial isolates from lower respiratory tract infection at HUCSH, 2019

Isolates	Pattern	Antibiotics (%)										
		CXT	TAT	AMP	CPR	COT	CLD	ERY	GN	PEN	CEF	CAF
***S. aureus*** **(*N*** **= 21)**	S	19 (90.6)	3 (14.3)		15 (71.4)	9 (42.9)	14 (66.7)	10 (47.6)	16 (76.2)	9 (45.0)		
	I	1 (4.7)	5 (23.8)	NR	1 (4.8)	2 (9.5)	0	0	3 (14.3)	2 (10.0)	NR	NR
	R	1 (4.7)	13 (61.9)		5 (23.8)	10 (47.6)	7 (33.3)	11 (52.4)	2 (9.5)	9 (45.0)		
***S. pneumoniae*** **(*N*** **= 9)**	S		2 (22.2)				8 (88.9)	6 (66.7)	NR	7 (77.8)		
	I	NR	1 (11.1)	NR	NR	NR	0	2 (22.2)		0	NR	NR
	R		6 (66.7)				1 (11.1)	1 (11.1)		2 (22.2)		
***S. pyogene*** **(*N*** **= 3)**	S		3 (100)	3 (100)			3 (100)	3 (100)	NR	3 (100)	2 (66.7)	
	I	NR	0	0	NR	NR	0	0		0	0	NR
	R		0	0			0	0		0	1 (33.3)	
***Enterococcus spp*** **(*N*** **= 1)**	S	NR	0	NR	1 (100)	0	1 (100)	0				0
	I		0		0	0	0	0	NR	NR	NR	0
	R		1 (100)		0	1 (100)	0	1 (100)				1 (100)
**Total (34)**	S	19 (90.6)	8 (23.5)	3 (100)	16 (72.0)	9 (40.0)	26 (76.5)	19 (55.9)	16 (76.2)	19 (57.6)	3 (100)	0
	I	1 (4.7)	6 (17.6)	0	1 (4.5)	2 (10.0)	0	1 (2.9)	3 (14.3)	2 (6.0)	0	0
	R	1 (4.7)	20 (58.8)	0	5 (22.7)	11 (50.0)	8 (23.5)	14 (41.2)	2 (9.5)	11 (33.3)	0	1 (100)

*NR* Not recommended, *CXT* Cefoxitin, *AMP* Ampicillin, *CEF* Cefepime, *TAT* Tetracycline, *CPR* Ciprofloxacin, *COT* Cotrimoxazole, *ERY* Erythromycin, *PEN* Penicillin, *CAF* Chloramphenicol, *CLD* Clindamycin, *GN* Gentamycin, *S* Sensitivity, *I* Intermediate, *R* Resistance

#### Gram negative bacteria

Gram-negative bacteria were sensitive to cefepime 93(86.0%), ciprofloxacin 84(77.8%), ampicillin 13(20.3%), augmentin 18(28.1%) and ceftazidime 49(45.4%). *K. pneumoniae* was sensitive to ciprofloxacin 33(91.7%), cefepime 30(83.3%) and cefoxitin 30(83.3%) and *K. pneumoniae was resistant* to augmentin 20(56.6%), ceftazidime 19(52.8%) and ampicillin 19(52.8%). *Pseudomonas* spp. *was sensitive* to cefepime 23(92.0%), ciprofloxacin 17(68.0%), gentamycin 17(68.0%), imipenem 16(64.0%) while *Pseudomonas* spp. *was* resistant to ceftazidime 17(68.0%). *E. coli* were susceptible to cefoxitin 18(81.1%), cefepime 17(77.3%) and show resistant to ampicillin 19(86.4%). *Acinetobacter* spp. was sensitive to cefepime 17(89.5%), ceftriaxone 16(84.2%), gentamycin 16 (84.2%) but resistant to cotrimoxazole 10(52.6%). All *Enterobacter* spp. and *Citrobacter* spp. were 100% sensitive to cefepime, tetracycline, ceftriaxone and ciprofloxacin antibiotics [Table 6].

**Table 6:** Antibiotic susceptibility pattern of gram-negative bacterial isolates of an adult patient with lower respiratory tract infection at HUCSH, 2019

Isolates	Pattern	Antibiotics (%)										
		CEF	CAZ	CXT	TAT	CTR	AMP	AUG	CPR	COT	GN	IMP
***K. pneumoniae*** **(*N*** **= 36)**	S	30 (83.3)	13 (36.1)	30 (83.3)	15 (41.7)	24 (66.7)	9 (25.0)	12 (33.3)	33 (91.7)	19 (52.8)		
	I	0	4 (11.1)	0	4 (11.1)	4 (11.1)	8 (22.2)	4 (11.1)	0	1 (2.8)	NA	NR
	R	6 (16.7)	19 (52.8)	6 (16.7)	17 (47.2)	8 (22.2)	19 (52.8)	20 (56.6)	3 (8.3)	16 (44.4)		
***Pseudomonas spp*** **(*N*** **= 25)**	S	23 (92.0)	6 (24.0)						17 (68.0)		17 (68.0)	16 (64.0)
	I	0	2 (8.0)	NR	NR	NR	NR	NR	0	NR	4 (16.0)	2 (8.0)
	R	2 (8.0)	17 (68.0)						8 (32.0)		4 (16.0)	7 (28.0)
***E.coli*** **(*N*** **= 22)**	S	17 (77.3)	11 (50.0)	18 (81.1)	3 (13.6)	14 (63.6)	3 (13.6)	5 (22.7)	13 (59.1)	10 (40.9)		
	I	1 (4.5)	2 (9.1)	2 (9.1)	5 (22.7)	0	0	1 (4.5)	0	1 (45.5)	NA	NR
	R	4 (18.2)	9 (40.9)	2 (9.1)	14 (63.6)	8 (36.4)	19 (86.4)	16 (72.7)	9 (40.9)	11 (4.5)		
***Acinetobacter*** **(*N*** **= 19)**	S	17 (89.5)	13 (68.4)			16 (84.2)			15 (78.9)	6 (31.6)	16 (84.2)	12 (63.2)
	I	0	1 (5.3)	NR	NR	2 (10.5)	NR	NR	1 (5.3)	3 (15.8)	2 (10.5)	2 (10.5)
	R	2 (10.5)	5 (26.3)			1 (5.3)			3 (15.3)	10 (52.6)	1 (5.3)	5 (26.3)
***Enterobacter spp*** **(*N*** **= 4)**	S	4 (100.0)	4 (100)	3 (75.0)	4 (100)	4 (100)	1 (25)	1 (25)	4 (100)	2 (50)		
	I	0	0	0	0	0	0	0	0	0	NA	NR
	R	0	0	1 (25.0)	0	0	3 (75)	3 (75)	0	2 (50)		
***Citrobacter spp*** **(*N*** **= 2)**	S	2 (100.0)	0	1 (50.0)	2 (100.0)	2 (100.0)	0	0	2 (100.0)	1 (50)		
	I	0	0	0	0	0	0	0	0	0	NA	NR
	R	0	2 (100.0)	1 (50.0)	0	0	2 (100.0	2 (100.0)	0	1 (50)		
**Total (*N*** **= 108)**	S	93 (86.0)	49 (45.4)	52 (81.3)	23 (35.9)	60 (72.3)	13 (20.3)	18 (28.1)	84 (77.8)	38 (45.8)	33 (75.0)	28 (63.6)
	I	1 (0.9)	9 (8.3)	2 (3.1)	10 (15.6)	6 (7.2)	8 (12.5)	5 (7.8)	1 (0.9)	5 (6.0)	6 (13.6)	4 (9.1)
	R	14 (13.0)	50 (46.3)	9 (15.6)	31 (48.4)	17 (20.5)	43 (67.2)	41 (64.0)	23 (21.3)	40 (48.2)	5 (11.4)	12 (27.3)

*NA* Not applied, *NR* Not recommended, *AUG* Augmentin, *CAZ* Ceftazidime, *CXT* Cefoxitin, *AMP* Ampicillin, *CEF* Cefepime, *TAT* Tetracycline, *CTR* Ceftriaxone, *CPR* Ciprofloxacin, *GN* Gentamycin, *IMP* Imipenem, *COT* Cotrimoxazole, *S* Sensitivity, *I* Intermediate, *R* Resistance

#### Multiple drug resistance (MDR) patterns of the isolates

In this study, the overall multidrug resistance (MDR) bacteria were 46/142(32.4%). *E. coli* was the most isolate that showed MDR 13(59.1%) followed by *S. aureus* 12(57.1%) and *K. pneumoniae* 12(33.3%). Majority of bacterial isolates were resistant to three classes of antibiotics 25(17.6%) [Table 7].

**Table 7:** multi-drug resistant rates of bacterial isolates among adult patient with lower respiratory tract infection at HUCSH, 2019

Isolate	R0(%)	R1(%)	R2(%)	R3(%)	R4(%)	R5(%)	≥R6(%)	Total (%)
*K. pneumoniae* (*N* = 36)	3 (8.3)	7 (19.4)	13 (36.1)	7 (19.4)	3 (8.3)	2 (5.6)	0	12 (33.3)
*S. aureus* (*N* = 21)	3 (14)	2 (9.5)	4 (19.0)	6 (28.6)	3 (14.3)	2 (9.5)	1 (4.7)	12 (57.1)
*E. coli* (*N* = 22)	3 (13.6)	3 (13.6)	4 (18.2)	3 (13.6)	3 (16.6)	0	6 (27.3)	12 (54.5)
*Pseudomonas spp* (*N* = 25)	4 (16.0)	7 (28.0)	10 (40.0)	4 (16.0)	0	0	0	4 (16.0)
*Acinetobacter spp* (*N* = 19)	6 (31.6)	5 (26.3)	6 (31.6)	2 (10.5)	0	0	0	2 (10.5)
*S. pneumoniae* (*N* = 9)	2 (22.2)	5 (55.5)	1 (11.1)	1 (11.1)	0	0		1 (11.1)
*Enterobacter spp* (*N* = 4)	0	1 (25.0)	2 (50.0)	1 (25.0)	0	0	0	1 (25.0)
*S. pyogene* (*N* = 3)	3 (100)	0	0	0	0	0		0
*Citrobacter spp* (*N* = 2)	0	0	1 (50.0)	1 (50.0)	0	0	0	1 (50.0)
*Enterococcus spp* (*N* = 1)	0	0	0	0	1 (100)	0	0	1 (100)
Total (*N* = 142)	24 (17.0)	30 (21.1)	41 (28.9)	25 (17.6)	10 (7.0)	4 (2.8)	7 (4.9)	46/142 (32.4)

Key: *R0* No antibiotic resistance, *R1* Resistance to one, *R2* Resistance to two, *R3* Resistance to three, *R4* Resistance to four, *R5* Resistance to five, and *≥ R6* Resistance to six and more than six antibiotics families

## Discussion

In this study, the overall of sputum culture positive of community acquired LRTI among adult patients was 136(33.5%). This finding is comparable to result reported from Arba Minch, Ethiopia [6] 40% but lower than Felege Hiwot [13] 40.3%, Jimma, Ethiopia [14], 45%, Zagazig, Egypt, 50.4% [15] and South India, 55.3% [16]. However, it was higher than studies result conducted in Central Kerala, India [17], Sri Lanka, Colombo [1], and Central Nepal [18], which were 26.34, 29.4 and 30.4% respectively. This difference may be due to geographical location, study period and socioeconomic status of the study population. The distribution of respiratory infections varies between populations and countries, depending on geographical, climatic and socioeconomic conditions [3].

*K. pneumoniae* 25.4% was the most frequent bacterial isolate followed by *Pseudomonas spp* 17.6%, *E. coli* 15.5% and *S. aureus* 14.8%. This finding is similar to the study conducted in Assuit, Egypt [19], in which *K. pneumoniae* 58% was the most frequent isolate followed by *P. aeruginosa* 28%. This finding also agrees with Janakpur, Nepal 27.0% [20], Zagazig, Egypt, 10.4% [15] and 31.5% South India [16], in which *K. pneumoniae* was the commonest detected of LRTI. In contrast, in the previous studies in Ethiopian such as Felege Hiwot 35.9% [13] Jimma 12.8% [14] and Arba Minch 11.8% [6] *S. pneumoniae* was the predominant bacterial pathogen of culture-positive sputum.

*S. aureus* which accounts 14.8% is higher than 8.8% Arba Minch [6] and 10.5% Jimma [14], but all most equal to Felege Hiwot [13] 14.4% in Ethiopian studies. When compared other parts of world studies, it is higher than Central India 3.55% [21], Zigazig, Egypt 4.7% [15] and Waziristan, Pakistan 5.9% [22]. However lower than the study conducted in Janakpur, Nepal 20.8% [20].

In our study, *K. pneumoniae* shows a high rate of sensitive to ciprofloxacin 91.7%, cefepime and cefoxitin 83.3% each and resistant to augmentin 56.6%, ceftazidime and ampicillin 52.8% each and tetracycline 47.2%. In contrast to this finding two other studies in Ethiopia showed, *K. pneumoniae* exhibit high rates of resistance to tetracycline 100% and augmentin 96.7% [13] and Regasa et al. reported as *K. pneumoniae* was 100% resistant to ampicillin and tetracycline [14].

The current study revealed that *S. aureus* was highly sensitive to cefoxitin 90.6%, gentamycin 76.2% and ciprofloxacin 71.4%. However, *S. aureus* was resistant to tetracycline 61.9%, cotrimoxazole 47.6% and penicillin 45%. In agreement to this, a study from Jimma, Ethiopia [14] reported *S. aureus* as high resistance to tetracycline 100%, penicillin and cotrimoxazole 81.3% to each, erythromycin 75%. Low resistance was observed for ciprofloxacin 31.3% and gentamycin 31.5%. In contrast to this high rates of sensitivity reported from Karachi, Pakistan to gentamicin 66.7% [23].

The overall magnitude of multidrug resistance (MDR) was 33.1% which is lower than other Ethiopian studies Jimma [14], Arba Minch [24] and Felege Hiwot [13] 60.3, 62.7 and 76% respectively. However, it is higher than Cairo, Egypt [25] 26.4%. This variation is may be due to the difference in the type of tested drugs and bacterial isolated. *E. coli* was the most isolate that showed MDR 59.1% followed by *S. aureus* 57.1% and *K. pneumoniae* 33.3% in this study. In a similar study in Cairo, Egypt [25] those organisms also exhibit MDR, *K. pneumoniae* 37.1%, *E. coli* 24.2%, *S. aureus* 14.5%.

Our study assessed the predisposing factors for culture-positive sputum sample among adult patient with LRTI. Accordingly, as compared to age > 65 years those with age group 18–35 years 3.856 times and age group 36–49 years 3.136 times at risk of culture-positive for LRTI. In contrast to our finding, most studies reported that elder age is a significant risk factor for LRTI, with the risk growing especially for people > 65 age [26, 27]. This difference is may be due to the 18–49 is working age and may have exposure to risk factor such as cigarette smoking, alcohol drinking and engaging in overcrowded area. In fact in developing countries, the main burden of hospitalized patients with *CAP* is among adults in the working-age but in developed countries in older patients with co-morbidity [28]. In this case, Ethiopia is one of the developing countries. Similarly, a study conducted on, risk factors for community-acquired pneumonia in adults: recommendations for its prevention, in Spain, state that age is a possible nonlinear effect, with older age as a risk factor for CAP [29].

Moreover, as compared to being single, those who are married 45% decreased for being culture-positive sputum of adult patient of LRTI. This may due to the single marital status individual at adult age have the exposure to alcohol drinking, cigarette smoking and chronic disease such as HIV. In fact, alcoholism-related conditions and comorbidities increased among single individual [30]. Similarly, the study from Spain [29], suggested that being married or in a partnership is a protective factor *for CAP* in comparison to being single, widowed or separated.

## Conclusion

*K. pneumoniae* was the predominant of lower respiratory tract infection pathogen followed by *Pseudomonas* species. Cefepime, ciprofloxacin and cefoxitin were the most effective antibiotics against gram-negative bacteria while clindamycin for gram-positive. However, the isolates showed resistance to common antibiotics ampicillin, augmentin, ceftazidime and tetracycline. Working age group and single marital status are associated with an increased risk of culture positive sputum of adult patient of lower respiratory tract infection. Therefore, culture and susceptibility test is vital for appropriate management of lower respiratory tract infection in the study area.

## Supplementary Information

**Additional file 1.**

## Data Availability

The datasets used and analyzed during the current study are available from the corresponding author on reasonable request.

## Abbreviations

AFB: Acid Fast Bacilli
ATCC: American Type Culture Collection
CAP: Community Acquired Pneumonia
CLSI: Clinical and Laboratory Standards Institute
HUCSH: Hawassa University Comprehensive Specialized Hospital
IRB: Institution Research Board
LRTIs: Lower Respiratory Tract Infections
MDR: Multi Drug Resistance
OPD: Out Patient Department
SPSS: Statistical Package for Social Science
