# Supplementary material for: Bacterial profile and drug susceptibility among adult patients with community acquired lower respiratory tract infection at tertiary hospital, Southern Ethiopia
Source: BMC Infect Dis. 2021 May 13;21:440. doi: 10.1186/s12879-021-06151-2 (PMC8120775; doi:10.1186/s12879-021-06151-2)
Supplement: Supplementary file 1 — Additional file 1. [file 12879_2021_6151_MOESM1_ESM.doc]

# Annex V: English Version of Questionnaire

**Code No _______**

Sociodemographic and associated factors

| No, | Questions | Coding categories | | Skip |
| --- | --- | --- | --- | --- |
| 1 | Sex | - 1. Male   2. Female | |  |
| 2 | How old were you at your last birthday? | ______________years | |  |
| 3 | Where is your origin of residence? | 1. Rural 2. Urban | |  |
| 4 | What was your marital status? | 1. Single 2. Married 3. Widowed 4. Divorced /separated | |  |
| 5 | What is your educational level? | 1. No formal education 2. Elementary 3. Secondary (9-12) 4. Diploma & above | |  |
| 6 | What is your occupation? | 1. Government employee 2. Private employee 3. Farmer 4. House wife 5. Student 6. No work | |  |
| 7 | What is your Family size? | ___________________ | |  |
| 8 | What your average monthly income in ETB? | 1. <1000 2. 1000-2000 3. 2001-3000 4. 3001-5000 5. >5000 | |  |
| 9 | Are you smoking a cigarette? | 1. Yes 2. No | |  |
| 10 | Are you drinking alcohol? | 1. Yes 2. No | |  |
| 11 | Have you heart disease? | 1. Yes 2. No | |  |
| 12 | Have you ever exposed to TB disease before two years? | 1. Yes 2. No | |  |
| 13 | Have you ever screened for HIV? | 1. Yes | 1. Negative 2. Positive |  |
|  |  | 1. No | |  |
